# Edible Biocomposite Films From Tragacanth and Apricot Gums Reinforced With Marine Algal Polysaccharides: Development, Characterization, and Antibacterial Efficacy

**DOI:** 10.1002/fsn3.72103

**Published:** 2026-07-10

**Authors:** Sahba Bahrami Freadooni, Leila Nateghi, Mansooreh Mazaheri, Ladan Rashidi, Mohammadreza Vafaee

**Affiliations:** ^1^ Department of Food Science and Technology, VaP.C. Islamic Azad University Varamin Iran; ^2^ Research Center of Food Technology and Agricultural Products Standard Research Institute (SRI) Karaj Iran; ^3^ Department of Biosystems Engineering, WT.C. Islamic Azad University Tehran Iran

**Keywords:** antimicrobial activity, fucoidan, TG‐ and PAGE‐based films

## Abstract

Increasing environmental concerns over petroleum‐based plastics have made the development of sustainable, functional food packaging a research priority. This study developed novel composite films by incorporating marine algal polysaccharides (MAPs) into matrices of either tragacanth gum (TG) or *Prunus armeniaca* L. gum (PAGE). The effects of incorporating purified fucoidan‐rich MAPs at different concentrations (0%, 0.5%, and 1% w/w) on the physicochemical, mechanical, barrier, structural, and antimicrobial properties of the films were systematically evaluated. Film thickness, opacity, solubility, water vapor permeability (WVP), tensile strength (TS), elongation at break (EB), thermal behavior, and morphological characteristics were analyzed to determine the influence of MAP incorporation. The results showed that specific formulations, particularly TG 1%–MAP 0.5% and PAGE 1%–MAP 0.5%, exhibited improved functional performance compared with other formulations. These films demonstrated increased UV‐blocking capacity while maintaining visible light transparency, enhanced thickness and tensile strength, and reduced WVP. Structural and thermal analyses suggested improved matrix organization and thermal resistance in MAP‐containing films, especially in the TG 1%–MAP 0.5% formulation. Antibacterial activity evaluated using the agar diffusion method showed measurable inhibition zones against *Escherichia coli* , *Staphylococcus aureus* , *Bacillus cereus* , and *Pseudomonas aeruginosa* , with the TG 1%–MAP 0.5% film exhibiting significantly larger inhibition zones of 20.00 ± 0.08 mm and 20.00 ± 0.32 mm against *E. coli* and *B. cereus* , respectively, compared to the PAGE‐based counterparts. Overall, the findings indicate that the incorporation of MAPs into TG and PAGE polysaccharide matrices can modify film structure and functional properties, highlighting their potential for sustainable food packaging applications.

## Graphical abstract

This study presents the fabrication of eco‐friendly biocomposite films via a solution casting method, utilizing a blend of marine algae polysaccharides (MAPs) with Tragacanth gum (TG) and *Prunus armeniaca* L. gum (PAGE). The integration of these biopolymers significantly optimizes film structural morphology, leading to superior mechanical performance characterized by increased tensile strength (TS) and reduced water vapor permeability (WVP). Furthermore, the developed films exhibit promising functional attributes, including effective UV‐blocking capability and antibacterial activity, confirming their viability as a sustainable and functional alternative in active food packaging applications.

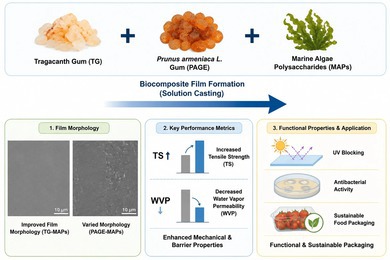

## 1 Introduction

Growing environmental concerns associated with the persistence of petroleum‐based plastics, together with increasing demand for safer and more sustainable food packaging, have intensified research on sustainable and functional film systems (James et al. 2024). Although synthetic polymers provide excellent mechanical strength and barrier performance, their limited degradability under natural conditions and long‐term environmental impact have accelerated the search for renewable alternatives. In this context, biopolymer‐based films derived from natural resources offer promising solutions, as they can reduce reliance on plastics while contributing to food preservation and quality maintenance (James et al. 2024). Consequently, the development of eco‐friendly and bio‐based films based on natural polymers has attracted substantial attention (Hadi et al. 2022; Rashidi 2022).

Among natural polymers, polysaccharides are particularly attractive due to their renewability, derived from natural resources, film‐forming ability, and oxygen barrier performance (Hadi et al. 2022; Tabassum et al. 2024). Polysaccharide‐based films generally exhibit good mechanical integrity and resistance to lipid transfer; however, their high sensitivity to moisture and variable structural stability under humid conditions often limit practical application (Tabassum et al. 2024). Recent studies have therefore focused on multicomponent and reinforced systems to improve mechanical, barrier, and functional properties. For example, Taktak and Kaya (2025a, 2025b) highlighted the importance of synergistic polymer interactions in improving film cohesion, structural stability, and overall functional performance, underscoring the need for rational composite design strategies.

Tragacanth gum (TG), a natural anionic polysaccharide obtained from *Astragalus* species, has emerged as a promising candidate for edible film development due to its emulsifying capacity, thickening behavior, and structural complexity (Hadi et al. 2022; Bahrami, Nateghi, and Rashidi 2025). TG consists primarily of tragacanthin (water‐soluble fraction) and bassorin (swelling, gel‐forming fraction), which together contribute to its rheological and film‐forming properties (Hadi et al. 2022). The presence of hydroxyl and carboxyl groups facilitates intermolecular interactions with other polymers and reinforcing components (Nejatian et al. 2020). Nevertheless, films based solely on TG often exhibit limited mechanical strength and suboptimal moisture barrier properties (Tabassum et al. 2024). To address these limitations, recent efforts have focused on composite systems incorporating TG with additional biopolymers or reinforcing agents to improve structural and functional performance (Bahrami, Nateghi, Rashidi, Nobandegani, and Ghorbanpour 2025; Bahrami, Nateghi, and Rashidi 2025). *Prunus armeniaca* L. gum exudate (PAGE) has also gained attention as a multifunctional hydrocolloid. Composed of approximately 82.79% carbohydrates and 1.36% protein, PAGE exhibits emulsifying and stabilizing capabilities comparable to gum Arabic due to its slight protein content and amphiphilic characteristics. PAGE has been applied as a fat replacer, stabilizer, and encapsulating agent in food systems (Gohari et al. 2024). Furthermore, composite films incorporating TG and other gums have shown improved functionality compared with single‐component matrices (Khodaei et al. 2020). Despite these promising attributes, PAGE has rarely been investigated as a structural component within complex multi‐polysaccharide film systems, particularly in combination with MAPs. Marine algae polysaccharides (MAPs), especially those derived from brown macroalgae, represent another important class of renewable biopolymers. These include fucoidan, alginate, laminarin, mannitol, and cellulose (Ummat et al. 2024). Fucoidan, characterized by a sulfated fucose‐rich backbone and a strong negative charge density, has attracted considerable interest for its biological activity and functional potential (Gohari et al. 2024). However, fucoidan alone lacks adequate film‐forming capacity and mechanical stability (Carpintero et al. 2023). Recent research has therefore explored alginate–fucoidan blends and related MAP systems to improve film performance (James et al. 2024). Nevertheless, integration of fucoidan‐rich MAPs with terrestrial exudate gums such as TG and PAGE remains largely unexplored.

Although individual components including TG, PAGE, and fucoidan‐rich MAPs have been investigated separately or in limited binary systems, a comprehensive evaluation of their combined incorporation into a unified composite film has not yet been reported. Considering their complementary structural features and shared anionic nature, the integration of TG (branched polysaccharide), PAGE (amphiphilic gum with minor protein content), and sulfated fucoidan may promote intermolecular interactions, network formation, and potentially complementary physicochemical and structural effects. It is therefore hypothesized that incorporating fucoidan‐rich MAPs into a TG‐PAGE matrix may improve mechanical strength, barrier performance, structural stability, and antimicrobial response compared with single‐ or binary‐component systems.

Accordingly, the present study aims to develop and characterize novel polysaccharide‐based composite films formulated from TG and PAGE integrated with fucoidan‐rich MAPs. The physicochemical, mechanical, barrier, antimicrobial, thermal, morphological, and structural properties of the developed films were systematically evaluated to assess their suitability as bio‐based food‐packaging materials under the conditions tested. Because biodegradability was not directly assessed in this work, no claims are made regarding environmental degradation performance.

## 2 Materials and Methods

### 2.1 Materials

Tragacanth gum (TG) with the average molecular weight (MW) of 8.4 × 10^5^ g/mol was purchased from a local store in Tehran (Iran) and *Prunus armeniaca* L. gum exudates (PAGE) were collected from apricot trees located at Jupar (a city in Mahan District, Kerman County, Kerman Province). Mueller Hinton Agar (MHA) was purchased from Sigma‐Aldrich (United States of America). All chemical materials used in the present study were of analytical grade.

### 2.2 Preparation of *Prunus armeniaca* L. Gum Exudates (PAGEs)

The collected PAGE was purified to remove impurities. Briefly, the crude gum was first dried at 115°C, ground, and passed through a 40‐mesh sieve to obtain a uniform powder. The gum powder was dissolved in distilled water and stirred at 25°C for 24 h. The solution was then stored at 4°C overnight to ensure complete hydration. Insoluble materials were removed by centrifugation at 3000 × *g* for 30 min. The soluble gum fraction was precipitated by adding ethanol at three times the weight of the gum. The precipitate was subsequently freeze‐dried, ground into powder, and stored in a sealed container until further use (Gohari et al. 2024).

The average molecular weight (MW) of the purified PAGE was determined to be 5.69 × 10^5^ g/mol using gel permeation chromatography (GPC) according to the method described by Gohari et al. (2024).

### 2.3 Preparation of Composite Films

The bioactive composite films were prepared using the solvent‐casting method (Tabassum et al. 2023). The films were formulated using TG at 0.5% and 1% (w/w) and purified MAPs rich in fucoidan at 0%, 0.5%, and 1% (w/w), obtained from our previous research (Bahrami, Nateghi, Rashidi, Nobandegani, and Ghorbanpour 2025; Bahrami, Nateghi, and Rashidi 2025). Likewise, MAPs at the same concentrations were combined with PAGE at 0.5% and 1% (w/w) to produce PAGE‐based films (Table 1). The selected concentration ranges were based on published film‐forming data for exudate gums (typically 0.5%–2% w/w) and on preliminary screening experiments conducted in our laboratory. Concentrations below 0.5% produced weak or discontinuous films, whereas levels above 1%–1.5% led to excessive viscosity during casting and heterogeneous film surfaces. MAPs were incorporated within the 0.5%–1% range because higher levels caused phase separation and brittleness, while lower levels did not provide noticeable functional enhancement. Thus, the chosen ratios represented the most stable and castable formulations while maintaining polymer homogeneity and desirable mechanical integrity. PAGE or TG solutions were heated to 90°C under magnetic stirring and subsequently cooled to 40°C before the addition of fucoidan‐rich MAPs. Glycerol (0.5% w/w) was added as a plasticizer. The glycerol level was selected based on literature for exudate‐ and polysaccharide‐based films, where 0.3%–1.5% typically balances flexibility and barrier performance, and on our preliminary screening. In our trials, ≤ 0.3% yielded brittle films with visible edge cracking and low elongation, while ≥ 1.0% caused tacky surfaces, reduced tensile strength, and increased water vapor permeability. A 0.5% level consistently produced smooth, homogeneous films with improved flexibility and handling without undermining strength or barrier properties. For film casting, a total dispersion volume of 100 mL was prepared for each formulation. The film‐forming dispersions were poured into standard polystyrene Petri dishes (90 mm diameter) and dried at 25°C for 24 h. The volume of dispersion added to each dish was adjusted to obtain uniform films. The dried composite films were carefully removed and conditioned at 23°C and 50% relative humidity in a desiccator before testing. Each formulation was produced in triplicate.

**TABLE 1:** Composite film formulations containing TG, PAGE, and MAPs (rich in fucoidan).

Treatments	TG‐based films	
	TG (%w/w)	MAPs (%w/w, dry basis)
TG 0.5%	0.5	0
TG 1%	1	0
TG 0.5% MAP 0.5%	0.5	0.5
TG 1% MAP 0.5%	1	0.5
TG 0.5% MAP 1%	0.5	1
TG 1% MAP 1%	1	1

Treatments	PAGE‐based films	
	PAGE (%w/w)	MAPs (%w/w, dry basis)
PAGE 0.5%	0.5	0
PAGE 1%	1	0
PAGE 0.5% MAP 0.5%	0.5	0.5
PAGE 1% MAP 0.5%	1	0.5
PAGE 0.5% MAP 1%	0.5	1
PAGE 1% MAP 1%	1	1

*Note:* It should be noted that the selected concentration levels (0.5% and 1% w/w) were chosen based on preliminary screening and literature guidance to ensure film‐forming capability and manageable viscosity during casting. While these levels allow comparative evaluation of formulation performance, they do not constitute a comprehensive dose–response design. A broader concentration range and additional intermediate levels would be required to establish detailed concentration‐dependent trends or perform systematic optimization.

### 2.4 Characterization of Films

#### 2.4.1 Transparency

The transparency of the nanocomposite films was determined according to the method described by Tabassum et al. (2024). Film specimens (1 × 3 cm) were placed in a spectrophotometric cell, and absorbance was measured in the 200–800 nm wavelength range using a UV–Vis spectrophotometer (Shimadzu UV‐1800, Japan). The transparency of the films was evaluated based on their absorbance values, where lower absorbance indicates higher film transparency.

#### 2.4.2 Water Barrier Properties

##### 2.4.2.1 Solubility

Film solubility was determined according to the method of Hadi et al. (2022), with minor modifications. Film samples (2 × 3 cm) were first conditioned in a desiccator at 0% relative humidity and weighed. They were then immersed in 80 mL distilled water under gentle agitation for 60 min at room temperature. After treatment, the samples were oven‐dried at 60°C until constant weight was achieved. Film solubility (%) was calculated using Equation (1):

(1) $$\text{Solubility}\;\left(\%\right)=\frac{\text{Initial}\;\mathrm{dry}\;\text{weight}\;\left(g\right)-\text{final}\;\mathrm{dry}\;\text{weight}\left(g\right)}{\text{Initial}\;\mathrm{dry}\;\text{weight}\;\left(g\right)}\times 100$$

##### 2.4.2.2 Water Vapor Permeability

The WVP of the films was measured gravimetrically using the desiccant method according to ASTM E96/E96M‐16 (Tabassum et al. 2024). Circular film specimens (9 cm diameter; test area 63.65 cm^2^) were sealed over the mouths of glass permeation cups containing 10 g of anhydrous calcium chloride (CaCl_2_, 0% RH). The cups were then placed in a climate‐controlled chamber maintained at 38°C ± 1°C and 90% ± 2% relative humidity (RH), creating a constant water vapor pressure gradient across the film. The weight of the cups was recorded every hour for 7 h using an analytical balance (±0.0001 g) to ensure a steady‐state weight. The weight change (Δ*m*) was plotted against time (Δ*t*), and the slope was determined by linear regression.

The WVP (g·m (m^−2^·s^−1^·Pa^−1^)) was calculated according to Equations (2) and (3):

(2) $$\text{WVTR}=\frac{\text{Slope}}{A}$$

(3) $$\mathrm{WVP}=\frac{\text{WVTR}\times L}{∆P}$$

where WVTR is the water vapor transmission rate (g m^−2^ s^−1^); 𝐴 is the exposed film area (m^2^); 𝐿 is the mean thickness of the film (m); and Δ𝑃 is the partial water vapor pressure difference across the film (Pa). Δ𝑃 was calculated as 𝑃_sat_ × (RH_2_−RH_1_), where 𝑃_sat_ is the saturation vapor pressure of water at 38°C (6624.5 Pa), RH_2_ is 0.90, and RH_1_ is 0.00. The *R* ^2^ values for all weight loss versus time regressions were greater than 0.99, indicating a steady‐state water vapor transmission (representative plots are provided in Figure S1). Each measurement was performed in triplicate.

#### 2.4.3 Thickness

The thickness of the prepared films was measured using a digital micrometer (accuracy: 0.001 mm; Mitutoyo, Japan). Five random points on each film were measured, and the average thickness value was determined. The mean thickness data were used for the evaluation of mechanical properties and water vapor permeability.

#### 2.4.4 Mechanical Properties

The mechanical properties, including TS and EB, were determined according to Khodaei et al. (2020). A Brookfield CT3 Texture Analyzer (Brookfield Engineering Laboratories Inc., USA) was employed. The prepared films were cut into 70 × 20 mm strips and conditioned in a climate chamber at 50% relative humidity (RH). Each strip was fixed between the grips of the texture analyzer and subjected to stretching at a rate of 1 mm/s. The initial gap between grips was 40 mm.

TS was calculated using Equation (4):

(4) $$\mathrm{TS}\;\left(\mathrm{MPa}\right)=\frac{l_{m}}{h\times 20\;\mathrm{mm}}$$

where *l*_m_ is the maximum force/load at break, *h* is the film thickness, and 20 mm represents the strip width. EB was calculated by dividing the film elongation at break by the initial gauge length (40 mm) and multiplying the value by 100.

### 2.5 Microstructure of Films

The surface morphology of the nanocomposite films was examined according to Zhang et al. (2023) with slight modifications. Observations were made using scanning electron microscopy (SEM; Hitachi, Japan) operated at an accelerating voltage of 10 kV. Film samples were fractured under liquid nitrogen, mounted on specimen plates, and sputter‐coated with gold under vacuum using an ion sputter coater. Images were captured at a magnification of 1000×.

### 2.6 Fourier Transform Infrared (FTIR) Analysis

FTIR spectra of the films were recorded using an FT‐IR spectrophotometer (PerkinElmer, USA) equipped with an attenuated total reflectance (ATR) accessory. Each spectrum was collected over the wavenumber range of 400–4000 cm^−1^ with a resolution of 2 cm^−1^. For each film type, three spectra were recorded at different positions on the film surface and averaged to ensure reproducibility. The spectra were baseline‐corrected and smoothed (Savitzky–Golay algorithm) prior to interpretation, following the method described by Zhang et al. (2023).

### 2.7 X‐Ray Diffraction (XRD) Analysis

The crystalline patterns of the nanocomposite films were analyzed using an X‐ray diffractometer (D8 Advance, Bruker, Germany) based on the method of Zhang et al. (2023) with slight modifications. Scans were performed at 35 kV and 30 mA using a Cu‐Kα radiation source. The diffraction angles (2*θ*) ranged from 5° to 40° at a scanning rate of 1.2°/min.

### 2.8 Differential Scanning Calorimetry (DSC) Analysis

The thermal behavior of the films was analyzed by differential scanning calorimetry (Model F3 200 DSC, Netzsch, Germany) following Shahvalizadeh et al. (2021). Approximately 10 mg of each film sample was sealed in an aluminum DSC pan, while an empty pan served as the reference. Heating was conducted from 0°C to 220°C at a rate of 10°C/min under a nitrogen atmosphere.

### 2.9 Antibacterial Analysis

The antimicrobial activity of the films was evaluated using the agar disk diffusion method (Shahvalizadeh et al. 2021) against Gram‐positive bacteria ( *Staphylococcus aureus* ATCC 6538, *Bacillus cereus* ATCC 11778) and Gram‐negative bacteria ( *Pseudomonas aeruginosa* ATCC 27853 and *Escherichia coli* ATCC 25922). MHA was prepared and poured into sterile Petri dishes according to the manufacturer's instructions. After inoculation with standardized bacterial suspensions, film disks (10 mm diameter) were placed on the agar surface. The plates were incubated at 37°C for 24 h, and the diameters of the inhibition zones surrounding each film were measured in millimeters (mm) to determine antibacterial activity.

### 2.10 Statistical Analysis

All experiments were conducted in triplicate, and data are presented as mean ± standard deviation. Statistical significance was evaluated using one‐way analysis of variance (ANOVA), followed by Duncan's multiple range test to compare means at a 95% confidence level (*p* < 0.05). Analyses were performed using SPSS software, version 2022.

## 3 Results and Discussion

### 3.1 Film Transparency

**FIGURE 1:**
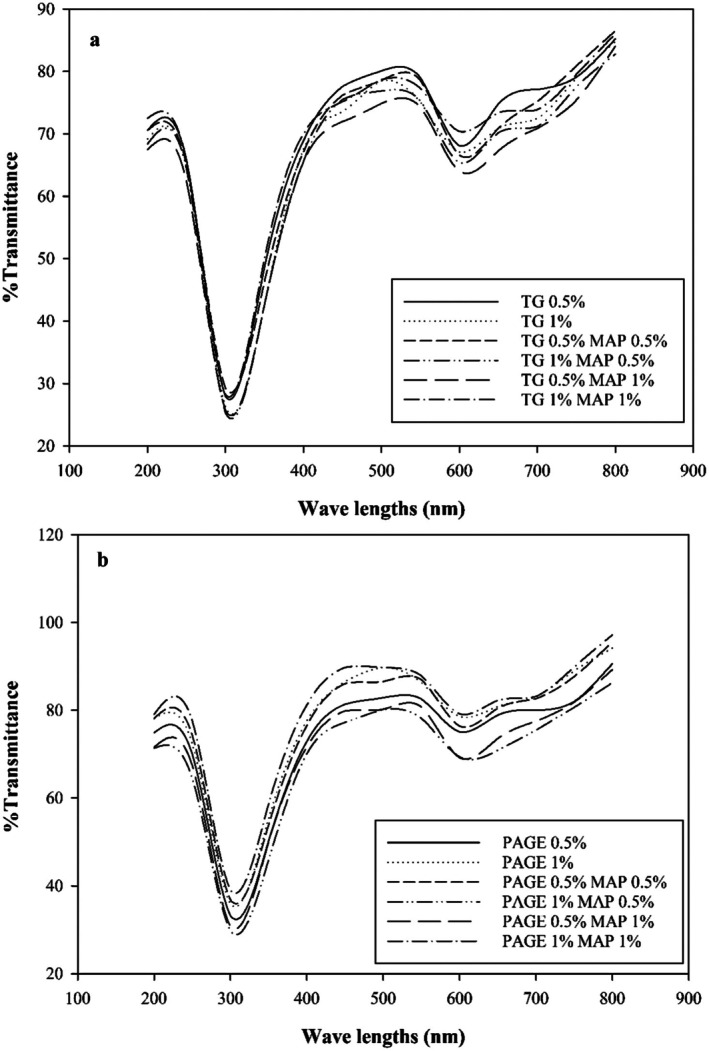
Transmittance spectra of (a) TG and (b) PAGE‐based films incorporated with MAPs rich in fucoidan.

The visual appearance of food packaging is critical for consumer acceptance. While transparency is often desirable, highly transparent films may permit excessive UV light transmission, potentially accelerating oxidative degradation in light‐sensitive foods. The transmittance spectra of the prepared films are shown in Figure 1a,b, and the corresponding numerical data at representative wavelengths are summarized in Table 2. Both TG‐ and PAGE‐based films exhibited a pronounced reduction in transmittance within the 200–350 nm range, corresponding to UV absorption by the polymeric components and incorporated MAPs. As shown in Table 2, the transmittance at 200 nm (*T*_200_) was near zero for all formulations, indicating an excellent barrier against high‐energy UV‐C radiation. Above this region, transmittance gradually increased up to 500 nm, followed by a slight characteristic dip between 500 and 600 nm (likely due to the presence of residual pigments in MAPs), and then rose toward 800 nm. This spectral profile indicates that the films effectively attenuate UV radiation while still allowing high transmission of visible light, achieving a balance between product protection and visual clarity.

**TABLE 2:** Light transmittance (%) at selected wavelengths and calculated opacity values of the MAP‐incorporated biocomposite films.

Sample	*T*_200_ (%)	*T*_400_ (%)	*T*_600_ (%)	*T*_800_ (%)	Opacity (mm^−1^)
TG 1% MAP 0.5%	0.2	68.4	66.1	84.1	1.51
TG 0.5% MAP 1%	0.1	71.2	64.2	83.1	2.13
PAGE 1% MAP 0.5%	0.3	72.1	76.2	89.3	0.91
PAGE 0.5% MAP 1%	0.2	78.1	69.2	86.3	1.48

To complement the spectral evaluation, opacity values were calculated at 600 nm and are presented in Table 2. The calculated opacity values confirmed the trends observed in the transmittance spectra. Within TG‐based systems, TG 1% MAP 0.5% and TG 0.5% MAP 1% exhibited the highest opacity values (1.51 and 2.13 mm^−1^, respectively) compared to control films. Overall, the incorporation of MAPs increased the opacity of both TG‐ and PAGE‐based films, which is consistent with the increased density and light‐scattering effect of the polysaccharide matrix upon the addition of MAPs. A reduction in transmittance and a corresponding increase in opacity in MAP‐fortified films can be attributed primarily to their natural pigment content, including fucoxanthin and chlorophylls. These pigments impart mild coloration and selective light absorption and are known contributors to the yellow‐brown appearance of crude fucoidan preparations, which has been associated with reduced transparency in nonpurified alginate systems (Roy et al. 2024; James et al. 2024). Additionally, TG inherently exhibits light scattering due to its colloidal, dispersive structure, which generates a whitish, light‐scattering network (Hadi et al. 2022; Mostafavi et al. 2016). When combined with MAPs, this intrinsic turbidity is further enhanced. This observation suggests that the final optical density is not merely a function of TG concentration, but rather a result of the combined light‐scattering effects from the colloidal bassorin particles in TG and the pigment‐rich polysaccharide chains of MAPs, which together form a more complex, light‐attenuating network.

Quantitatively, TG 1% MAP 0.5% and TG 0.5% MAP 1% films exhibited minimum UV transmittance values of 25.7% and 25.3%, respectively, with corresponding visible light transmittance values of 84.0% and 82.8% at 800 nm. Similarly, PAGE 1% MAP 0.5% and PAGE 0.5% MAP 1% films displayed minimum UV transmittance values of 30.7% and 29.7%, with visible light transmittance values of 89.3% and 86.3% at 800 nm, respectively. These results confirm that the incorporation of fucoidan‐rich MAPs enables controlled opacity and enhanced UV light attenuation, especially in the UV‐B region, without compromising the visual appearance of the films. Overall, TG‐ and PAGE‐based biocomposite films containing MAPs demonstrate favorable optical characteristics, combining functional protection of light‐sensitive foods with an esthetically acceptable level of transparency. These properties highlight their potential for sustainable food‐packaging applications. The optical performance of the prepared films was contextualized by comparing our findings with values reported for similar polysaccharide‐based matrices in the literature. Our results for UV‐C barrier properties ($T_{200}\approx 0\%$) are consistent with previous reports on fucoidan‐ and alginate‐based films, where incorporation of bioactive compounds effectively suppressed high‐energy UV transmission (Roy et al. 2024; James et al. 2024). Furthermore, the observed opacity values (0.91–2.13 mm^−1^) fall within the range typically reported for active biocomposite packaging materials, which often range from 0.5 to 3.0 mm^−1^ (Hadi et al. 2022; Mostafavi et al. 2016). The slight reduction in visible‐light transparency compared to pure biopolymer films is a common trade‐off observed in functionalized active packaging, yet the films maintained an acceptable level of clarity for consumer acceptance, highlighting their potential as sustainable alternatives for light‐sensitive food applications.

### 3.2 Solubility

The water solubility of bio‐based films prepared from natural biopolymers and hydrocolloids serves as a key indicator of their moisture resistance and structural integrity. As presented in Table 3, for the TG‐based films, the lowest solubility values were obtained for TG 1% MAP 0.5% (22.00% ± 0.23%) and TG 0.5% MAP 1% (23.00% ± 0.40%), whereas the highest value corresponded to TG 1% MAP 1% (34.00% ± 0.21%), followed by TG 0.5% MAP 0.5% (31.00% ± 0.16%). These findings suggest that the water solubility behavior is governed by a complex interaction between TG and MAP concentrations rather than the concentration of a single component. In control films, increasing the TG concentration from 0.5% to 1% led to a significant rise in solubility from 25.00% to 28.00% (*p* < 0.05). However, a contrasting trend was observed upon the incorporation of MAPs. Specifically, when 0.5% MAP was present, increasing the TG level from 0.5% to 1.0% caused the solubility to drop markedly from 31.00% to 22.00%. This reduction is likely attributed to the increased presence of the bassorin fraction in TG, a semi‐crystalline component that remains insoluble in water (Tabassum et al. 2024), which in combination with MAP molecules, appears to promote a more stable and less water‐sensitive matrix. The relatively lower solubility detected in TG 1% MAP 0.5% compared to TG 0.5% MAP 0.5% implies that specific intermolecular interactions between TG and MAP restricted water diffusion. A more favorable arrangement of hydrogen‐bonding interactions at these specific ratios may have contributed to stabilization of the polymer matrix (Brovko et al. 2024). Consequently, the stability of the matrix against water dissolution should be interpreted as a synergistic phenomenon; while the insoluble bassorin provides a physical barrier, the specific ratio of MAP likely optimizes the intermolecular hydrogen bonding, thereby anchoring the polymer chains more effectively than in the single‐component formulations.

**TABLE 3:** Solubility values of composite films containing TG, PAGE, and MAPs.

Treatments	Solubility of TG‐based films (%)
TG 0.5%	25.00 ± 0.34^h^
TG 1%	28.00 ± 0.27^f^
TG 0.5% MAP 0.5%	31.00 ± 0.16^e^
TG 1% MAP 0.5%	22.00 ± 0.23^j^
TG 0.5% MAP 1%	23.00 ± 0.40^i^
TG 1% MAP 1%	34.00 ± 0.21^c^

Treatments	Solubility of PAGE‐based films (%)
PAGE 0.5%	28.00 ± 0.43^f^
PAGE 1%	32.00 ± 0.38^d^
PAGE 0.5% MAP 0.5%	30.00 ± 0.05^b^
PAGE 1% MAP 0.5%	25.00 ± 0.40^h^
PAGE 0.5% MAP 1%	27.00 ± 0.34^g^
PAGE 1% MAP 1%	37.00 ± 0.42^a^

*Note:* Different superscript letters indicate significant differences (*p* < 0.05).

Earlier research demonstrated that alginate–fucoidan systems can form interpolymeric complexes stabilized by hydrogen bonding and hydrophobic associations. A comparable mechanism appears operative in TG 0.5% MAP 1%, where the elevated MAP content significantly reduced the film's solubility to 23.00%. Collectively, formulations containing either higher TG with moderate MAP or lower TG with higher MAP percentages revealed lower solubility than “double high” (1% TG, 1% MAP) or “double low” (0.5% TG, 0.5% MAP) combinations. This suggests that a balanced ratio of these components promotes optimal cross‐linking density and structural compactness. Conversely, the higher solubility observed in TG 0.5% MAP 0.5% and TG 1% MAP 1% films (31.00% and 34.00%, respectively) might be associated with a less efficient arrangement of the polymer network, where the specific proportions of the components may not have been optimal to effectively shield hydrophilic sites from water interaction.

For PAGE‐based films, the greatest solubility was found in PAGE 1% MAP 1% (37.00%), followed by PAGE 1% (32.00%) and PAGE 0.5% MAP 0.5% (30.00%), whereas PAGE 1% MAP 0.5% (25.00%) and PAGE 0.5% MAP 1% (27.00%) had the lowest values. Consistent with the trends in TG‐based systems, these outcomes confirm that solubility is governed by the synergistic interaction between the polymer matrix and MAP concentration. Overall, PAGE‐based films tended to be more soluble than their TG‐based counterparts (*p* < 0.05), likely because PAGE lacks an insoluble fraction analogous to bassorin, making it inherently more hydrophilic.

In agreement with prior studies, the addition of TG to aloe vera–alginate systems lowered solubility through the contribution of bassorin microdomains (Hadi et al. 2022). Likewise, the presence of fucoidan has been reported to decrease solubility in alginate–chitosan films (Gomaa et al. 2018). Therefore, the current results corroborate that the moisture resistance of these films is governed by the interplay between the structural components. The presence of bassorin microdomains in TG, when complemented by the functional groups of MAPs, effectively limits water penetration. This confirms that the barrier efficiency is a product of the integrated biocomposite structure rather than the isolated contribution of TG or MAP alone (Hadi et al. 2022; Tabassum et al. 2024).

Given these observations, TG 1% MAP 0.5%, TG 0.5% MAP 1%, PAGE 1% MAP 0.5%, and PAGE 0.5% MAP 1% formulations were selected for further characterization owing to their favorable combination of low UV permeability, high visible transparency, and minimal water solubility.

### 3.3 Water Vapor Permeability (WVP)

Based on the selection criteria mentioned above, TG‐based formulations (TG 1% MAP 0.5% and TG 0.5% MAP 1%) and PAGE‐based formulations (PAGE 1% MAP 0.5% and PAGE 0.5% MAP 1%) were chosen for further characterization. These films were selected to represent samples exhibiting high optical transparency and low solubility. It should be noted that the present formulation design does not constitute a full factorial experimental matrix in which TG/PAGE and MAP concentrations are independently varied. Consequently, the observed variations in film thickness and WVP reflect the combined influence of formulation composition rather than the isolated contribution of each component. A systematic factorial or response surface methodology (RSM) design would enable clearer quantification of individual and interaction effects and is therefore recommended for future optimization studies. According to the WVP data presented in Table 4, significant differences were observed among the selected formulations (*p* < 0.05). The lowest permeability value was obtained for TG 1% MAP 0.5%, while TG 0.5% MAP 1% exhibited a slightly higher but still relatively low WVP. A similar pattern was observed for PAGE‐based films, where PAGE 1% MAP 0.5% demonstrated a lower WVP than PAGE 0.5% MAP 1% (*p* < 0.05). Overall, the TG‐based films showed significantly lower permeability compared to PAGE‐based ones (*p* < 0.05). This trend is consistent with the solubility results, suggesting that PAGE‐based films may permit greater water vapor transmission, whereas TG‐containing formulations tend to provide more effective resistance to moisture diffusion. The presence of the insoluble bassorin fraction in TG has been reported to influence the barrier properties of TG‐based films (Hadi et al. 2022). Previous studies have also shown that the incorporation of fucoidan into alginate matrices can increase WVP, particularly at higher additive concentrations (James et al. 2024). In contrast, the addition of *S. pallidum* polysaccharide to chitosan films was reported to decrease WVP due to enhanced intermolecular interactions that act as barriers to water vapor transfer (Zhang et al. 2023). In the present study, the lower permeability observed in TG‐containing films may be associated with the formation of a relatively dense polymer network and reduced molecular mobility within the matrix. While the abundance and spatial arrangement of hydroxyl (−OH) groups typically influence permeability, the lower WVP values observed in this study are likely driven by a synergistic interaction between the TG and MAP components. This synergy, combined with the high molecular weight of TG and the presence of L‐fucose side chains, promotes a more integrated polymer matrix. Consequently, the intermolecular associations between the TG and MAP chains reduce the free volume and enhance the tortuosity of the diffusion path for water vapor (Khodaei et al. 2020). It is important to clarify that although TG contains hydrophilic groups capable of interacting with water, water vapor permeability is governed not only by water affinity but also by the structural organization of the polymer matrix. In other words, moisture sorption and vapor diffusion are related but not identical phenomena. A film may absorb some moisture due to polar functional groups, yet still exhibit relatively low WVP if its matrix is dense, cohesive, and presents a tortuous path for vapor transport. In the present study, the lower WVP of TG‐based films may therefore reflect the combined effect of bassorin‐containing microdomains, higher matrix compactness, and favorable intermolecular associations with MAP, which together restrict vapor diffusion despite the hydrophilic nature of the polysaccharide components.

**TABLE 4:** Water vapor permeability and thickness of composite films containing TG, PAGE, and MAPs (rich in fucoidan).

Sample	WVP (g·m/(m^2^·s·Pa))	Thickness (μm)
TG 1% MAP 0.5%	0.40 ± 0.00^d^	119.00 ± 3.99^b^
TG 0.5% MAP 1%	0.50 ± 0.01^c^	90.00 ± 3.01^d^
PAGE 1% MAP 0.5%	0.70 ± 0.04^b^	129.00 ± 2.57^a^
PAGE 0.5% MAP 1%	0.90 ± 0.01^a^	107.00 ± 2.31^c^

*Note:* Different small superscripts indicate significant difference between the columns (*p* < 0.05).

### 3.4 Thickness

The thickness values of the selected films are presented in Table 4. For the TG‐based formulations, thickness ranged between 90 and 119 μm, whereas the PAGE‐based films exhibited slightly higher values ranging from 106 to 129 μm. For both TG‐ and PAGE‐based systems, films prepared at 1% (w/w) TG or PAGE generally exhibited greater thickness than those prepared at 0.5% (w/w), which is consistent with the higher total gum content and greater solids loading during casting. Within each gum type, however, the incorporation of MAP at 0.5% or 1% (w/w) did not consistently increase thickness and, in some cases, was associated with slightly thinner films (*p* < 0.05). This suggests that, at the relatively low MAP levels employed, the marine polysaccharide phase may influence the packing and arrangement of the polymer network during drying rather than simply contributing additively to the solids content. It is important to note that any apparent inverse relationship between thickness and WVP should not be interpreted as an independent experimental finding demonstrating improved barrier properties. Because WVP is calculated using film thickness as a parameter, an apparent inverse dependence on thickness is partly a mathematical consequence of the standard WVP equation and does not, by itself, indicate enhanced barrier performance. Therefore, thickness and WVP are considered separately in our interpretation: thickness values describe the physical dimensions of the films, while WVP reflects the intrinsic ability of the polymer matrix to resist water vapor transmission under the specified test conditions. Within this framework, the lower WVP values observed in TG‐based films compared with PAGE‐based ones may be related to differences in matrix structure or hydrophilicity between the two film systems. The observed differences in thickness and WVP among the formulations reflect the combined influence of composition rather than isolated component effects, given the nonfactorial design of the study. The thickness values obtained in this study are within the representative range reported for polysaccharide‐based edible films, typically between 50 and 150 μm (Tabassum et al. 2024; Hadi et al. 2022; Pouralkhas et al. 2023), although direct mechanistic comparison is limited by differences in polymer type and casting conditions (Da Silva et al. 2025). Consistent with general trends in film casting, increasing the primary gum content (TG or PAGE) tended to produce thicker films, while the subtle variations seen with MAP suggest altered network arrangements or packing density. These compositional variations influence the final film microstructure and, in turn, affect their water vapor barrier performance (Fan et al. 2025).

### 3.5 Tensile Strength and Elongation at Break

The mechanical properties, including tensile strength (TS) and elongation at break (EB) of the selected films, were evaluated, and the results are summarized in Table 5. TS reflects the film's resistance to rupture under stress, while EB corresponds to its ability to undergo deformation before breaking (Tabassum et al. 2024).

**TABLE 5:** Tensile strength and elongation at break (%) of composite films containing TG, PAGE, and MAPs (rich in fucoidan).

Sample	Tensile strength (MPa)	Elongation at break (%)
TG 1% MAP 0.5%	60.00 ± 0.34^a^	11.00 ± 0.19^c^
TG 0.5% MAP 1%	50.20 ± 1.16^b^	29.00 ± 0.25^a^
PAGE 1% MAP 0.5%	35.60 ± 2.00^c^	17.00 ± 0.85^b^
PAGE 0.5% MAP 1%	28.00 ± 0.95^d^	5.80 ± 0.53^d^

*Note:* Different small superscripts indicate significant difference between the columns (*p* < 0.05).

The TG‐based composite films exhibited significantly higher TS values than the PAGE‐based films (*p* < 0.05). The TS values for TG‐based films ranged from 50 to 60 MPa, whereas those for PAGE‐based films were lower, between 27 and 35 MPa. Moreover, increasing the concentration of TG or PAGE from 0.5% to 1% (w/w) resulted in higher TS values (*p* < 0.05). These results suggest that the primary hydrocolloids play a central role in establishing the structural framework of the films.

The addition of MAPs rich in fucoidan at higher concentrations led to a reduction in TS, suggesting a possible disruption of the cohesive network between hydrocolloid chains. The formulations TG 1% MAP 0.5% and TG 0.5% MAP 1% recorded TS values of 60.01 MPa and 50.22 MPa, respectively, while PAGE 1% MAP 0.5% and PAGE 0.5% MAP 1% exhibited lower values of 35.62 and 27.54 MPa, respectively. The reduction in TS at higher MAP levels may be due to increased free volume and less efficient packing of polymer chains, which could reduce matrix cohesion. In contrast, the superior TS of TG‐based films can be attributed to the presence of bassorin, the water‐insoluble fraction of TG, which enhances hydrophobic associations and network rigidity. Within this context, the mechanical strengthening observed is likely a result of synergistic reinforcement between the TG matrix and MAP components, where the insoluble bassorin fraction and the marine polysaccharides create a more cohesive and rigid network that limits polymer chain mobility. These possible intermolecular associations may have contributed to improved stress‐bearing capacity of TG‐based films compared with PAGE‐based ones.

Consistent with these findings, Hadi et al. (2022) reported that TG incorporation enhanced both tensile strength and flexibility in alginate/aloe vera composite films, confirming the reinforcing capability of TG as a structural modifier in biopolymer networks. Accordingly, when TG was incorporated into alginate/aloe vera composite films, the TS increased markedly from 20.92 MPa in the control film to 67.49 MPa. This enhancement can be attributed to the synergistic interactions between TG and alginate polymer chains that strengthened the internal cohesion of the matrix. Similarly, Pirsa et al. (2020) reported that the increase in TS resulted from TG molecules intercalating between starch chains, which reinforced van der Waals forces among polymer molecules, leading to higher mechanical strength. Conversely, the introduction of seaweed polysaccharides into chitosan films has been shown to reduce TS, likely due to steric hindrance that limits effective polymer–polymer interactions (Zhang et al. 2023). A related observation was noted by Pouralkhas et al. (2023), where the addition of fucoidan significantly reduced the TS of gelatin films, falling from 29.27 MPa (control) to 4.32 MPa for the film containing 2.5% fucoidan. In contrast, the incorporation of 0.5%–2% TG into whey protein composites did not notably affect TS (*p* > 0.05) (Tonyali et al. 2018).

Regarding elongation at break (EB), results presented in Table 5 show that TG 0.5% MAP 1% had the highest EB value (28.93%), followed by PAGE 1% MAP 0.5% (16.56%), TG 1% MAP 0.5% (10.74%), and PAGE 0.5% MAP 1% (5.81%). The addition of MAPs produced different effects depending on the base polymer. In the TG‐based formulations, increasing MAP concentration to 1% while reducing TG to 0.5% enhanced EB, indicating a synergistic interaction that improved flexibility. In contrast, for PAGE‐based films, increasing MAP content led to lower EB, suggesting that at these specific ratios, the interaction between MAP and PAGE may not promote the same degree of plasticization or network flexibility as seen in TG‐based systems.

The findings align with previous studies showing that TG improved the flexibility of aloe vera and alginate films (Hadi et al. 2022). In their work, EB increased from 0.7% (control) to 5.5% when a high TG concentration was employed. This behavior was attributed to TG's gel‐forming capacity and water absorption ability, which contributed to its plasticizing effect within the film matrix. Conversely, fucoidan incorporation into chitosan films reduced EB due to a decrease in intermolecular hydrogen bonding and the introduction of hydrophilic groups that weakened polymer cohesion (Liang et al. 2024). Similar negative effects on flexibility were observed when seaweed polysaccharides were added to chitosan systems, where steric hindrance was identified as the major factor for reduced EB (Zhang et al. 2023).

Interestingly, fucoidan addition to gelatin‐based films produced different outcomes; EB increased from 47.31% (control) to 58.53% at 2.5% fucoidan, but subsequently decreased to below 50% at higher fucoidan levels (Pouralkhas et al. 2023). In whey protein–TG composites, EB increased significantly when 1% TG was incorporated (Tonyali et al. 2018), rising from 46.25% (control) to 120%, implying that TG disrupted protein–protein interactions and created additional free volume within the matrix. However, excessive TG (1.5%–2%) caused a sharp decline in EB (85% and 69.25%, respectively), possibly due to the saturation of free spaces by excess TG and the resulting densification of the polymer network.

Collectively, among the formulations examined, TG 1% MAP 0.5% and PAGE 1% MAP 0.5% were selected for subsequent analyses due to their balanced performance in terms of tensile strength, thickness, and moisture barrier properties, reflecting the most effective synergistic integration of the biopolymer components within the constraints of the study.

### 3.6 Microstructure

**FIGURE 2:**
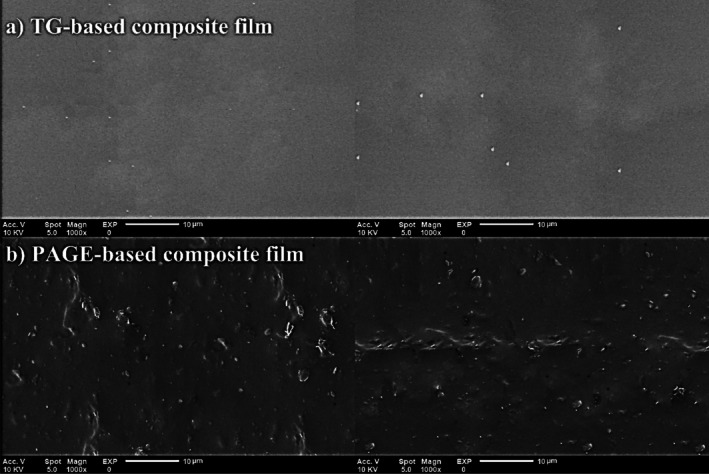
Scanning electron microscopy of “TG 1% MAP 0.5%” (a) and “PAGE 1% MAP 0.5% (b)”.

The surface microstructure of the composite films was examined using scanning electron microscopy (SEM) to evaluate the structural integrity and component distribution within the polymer matrices (Figure 2a,b). To provide a representative comparison of the two primary hydrocolloid systems (TG and PAGE) under optimal performance conditions, formulations TG 1% MAP 0.5% and PAGE 1% MAP 0.5% were selected for analysis. These specific formulations were chosen because they exhibited the highest tensile strength, appropriate thickness, and favorable water vapor barrier properties within their respective groups, thereby representing the most cohesive and structurally stable networks formed in this study. As shown in Figure 2a, the TG 1% MAP 0.5% film exhibited a relatively smooth and semi‐homogeneous surface with a few small, isolated micro‐domains. This uniform morphology is consistent with the higher tensile strength observed for this formulation and aligns with morphological features previously reported for TG‐based matrices, where the bassorin fraction contributes to a dense and continuous network (Khodaei et al. 2020). In contrast, the PAGE 1% MAP 0.5% film displayed a comparatively rougher and more heterogeneous surface topography (Figure 2b). The increased surface irregularities in the PAGE‐based system may reflect a different degree of interfacial compatibility between the apricot gum and the marine polysaccharides compared to the TG‐based system. Similar differences in surface morphology between various polysaccharide‐based films have been documented in previous studies (Gomaa et al. 2018).

Despite these surface variations, both films appeared continuous without large cracks or structural discontinuities, suggesting the formation of coherent matrices suitable for film applications. However, at the magnification used, SEM observations provide qualitative information on surface topography; further molecular‐level analyses would be required to fully elucidate the specific phase behaviors or intermolecular arrangements responsible for the observed mechanical and barrier performance.

### 3.7 Chemical Structure

**FIGURE 3:**
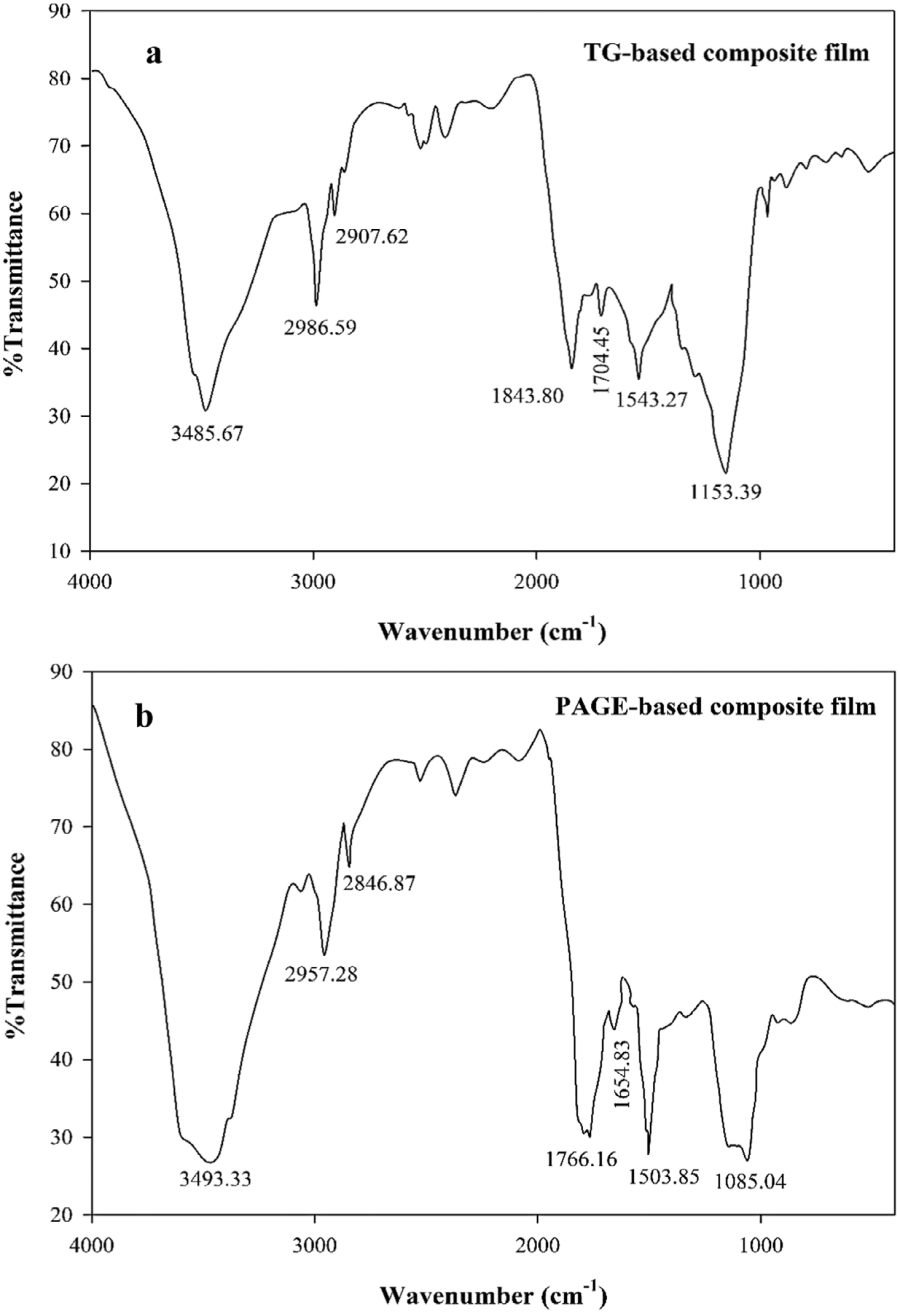
Fourier transform infrared spectroscopy (FTIR) of “TG 1% MAP 0.5%” (a) and “PAGE 1% MAP 0.5% (b)”.

The FTIR spectra of the selected TG‐ and PAGE‐based films recorded over the wavenumber range of 4000–400 cm^−1^ are presented in Figure 3a,b. Figure 3a shows the spectrum of the TG 1% MAP 0.5% film. A broad absorption band at 3485 cm^−1^ was attributed to the stretching vibrations of hydroxyl (–OH) groups. This band represents the combined contribution of free, intermolecular, and intramolecularly bound hydroxyl groups, indicating hydrogen bonding within the polysaccharide matrix and with water molecules (Sheybani et al. 2023a, 2023b; Khodaei et al. 2020). In comparison, the PAGE‐based film (Figure 3b) exhibited the corresponding broad band at 3493 cm^−1^. The shift of the O–H band toward a slightly higher wavenumber in the PAGE‐based film, together with the apparent difference in band shape, suggests variation in the hydrogen‐bonding environment between the two film systems.

Additional peaks positioned at 2986 and 2907 cm^−1^ in the TG‐based film are associated with C–H stretching of methyl groups, predominantly originating from the bassorin fraction in TG‐based systems (Khodaei et al. 2020; Mostafavi et al. 2016). Similar peaks have been previously reported in seaweed polysaccharides and PAGE‐derived films (Gomaa et al. 2018; Sharma et al. 2020). Peaks at 2926–2927 cm^−1^ correspond to C–H stretching and bending vibrations (Sharma et al. 2020; Sheybani et al. 2023a, 2023b), while bands in the 2925–2940 cm^−1^ and 2856–2867 cm^−1^ ranges are linked to asymmetric and symmetric CH_2_ stretching, respectively (Nejatian et al. 2020). In the PAGE‐based film, peaks detected near 2957 and 2846 cm^−1^ were assigned to C–H vibrational modes of methyl and methylene groups, likely stemming from proteinaceous residues in PAGE, in agreement with findings for other tree exudate gums such as gum acacia (Gohari et al. 2024). The differences in ‐peak position between the TG‐ and PAGE‐based films indicate differences in the local chemical environment of these aliphatic groups.

Within the TG 1% MAP 0.5% spectrum (Figure 3a), a band at 1843 cm^−1^ was observed. Although this region has been associated with carbonyl‐related vibrations in some studies (Nejatian et al. 2020; Rezvankhah et al. 2022), the position of this band is relatively uncommon for typical polysaccharide‐based films and therefore should be interpreted with caution. In contrast, the PAGE‐based film exhibited distinct bands at 1766 cm^−1^ and 1654 cm^−1^, which are attributed to C=O stretching in protein‐related structures, while the band at 1503 cm^−1^ corresponds to amide vibrations (Liang et al. 2024). The differences in this spectral region suggest that the TG‐ and PAGE‐based matrices provide different chemical surroundings for carbonyl‐containing groups and associated interactions.

An amide II band was identified around 1543 cm^−1^ in the TG‐based film (Rezvankhah et al. 2022). A prominent peak at 1153 cm^−1^ arises from C–O stretching and, possibly, S=O vibrations within sulfated polysaccharides such as fucoidan (Bahrami, Nateghi, Rashidi, Nobandegani, and Ghorbanpour 2025; Puhari et al. 2022; Samani et al. 2023). This feature reflects the high glycosidic linkage density of TG (Khodaei et al. 2020). Additional peaks observed between 1100–1500 cm^−1^ are characteristic of C–O, C–O–C, and C–N stretching vibrations in complex sulfated marine polysaccharides (Bahrami, Nateghi, Rashidi, Nobandegani, and Ghorbanpour 2025; Samani et al. 2023). The incorporation of fucoidan has been reported to influence hydroxyl absorbance and hydrogen‐bonding patterns within composite films (Samani et al. 2023). In the PAGE‐based film, a characteristic peak at 1085 cm^−1^ denotes S=O stretching vibrations, −supporting the presence of sulfated polysaccharides such as fucoidan within the film matrix (Liang et al. 2024). The shift of this sulfate‐related band from 1153 cm^−1^ in the TG‐based film to 1085 cm^−1^ in the PAGE‐based film suggests differences in the local chemical environment and interaction of the sulfated groups within the two polymer networks.

### 3.8 Crystallinity Structure

**FIGURE 4:**
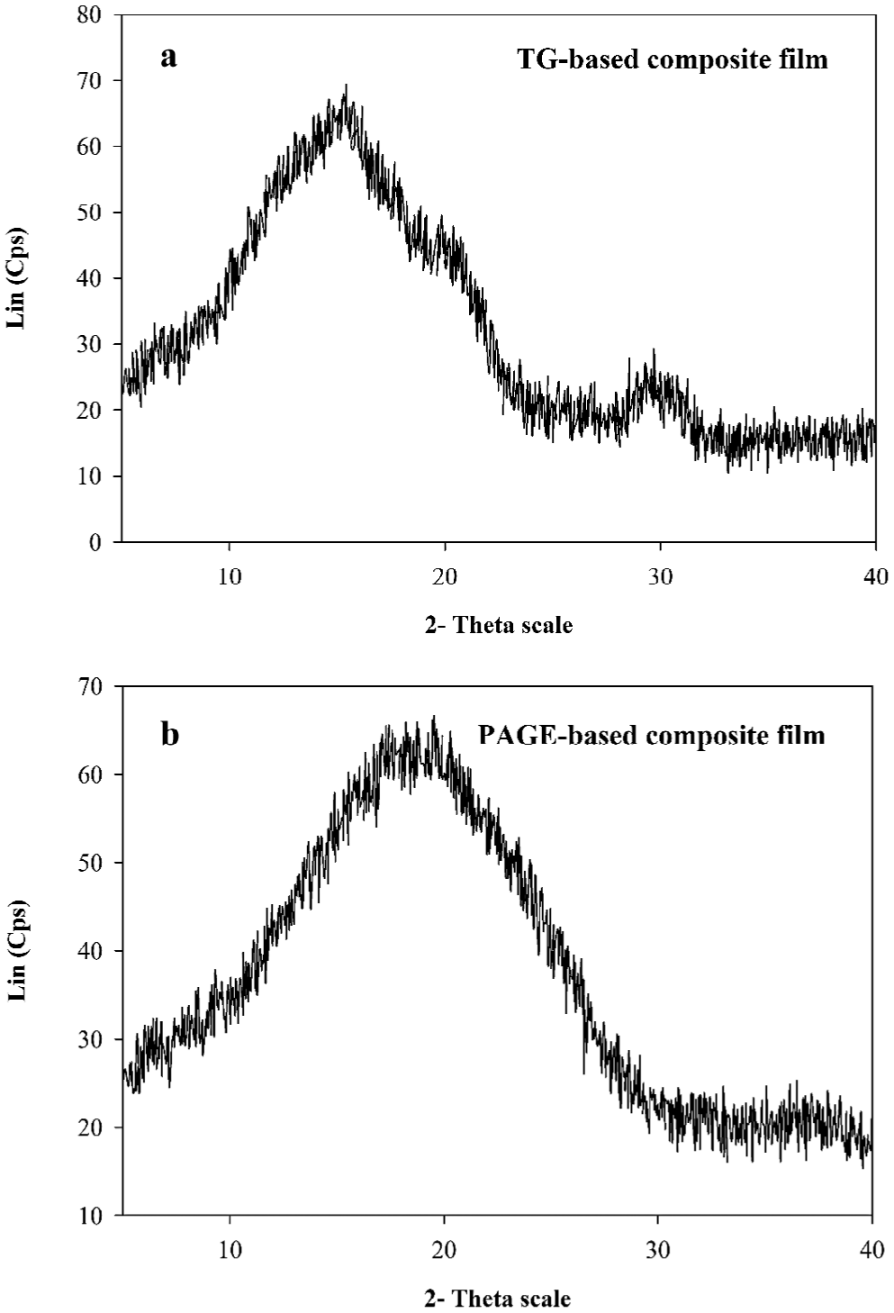
X‐ray diffractions (XRD) of “TG 1% MAP 0.5%” (a) and “PAGE 1% MAP 0.5% (b)”.

The X‐ray diffraction (XRD) patterns of TG‐ and PAGE‐based films incorporating MAPs (rich in sulfated polysaccharides such as fucoidan) are presented in Figure 4a,b. The diffraction patterns of both TG 1% MAP 0.5% and PAGE 1% MAP 0.5% films are dominated by broad halos, which are typical of largely amorphous polysaccharide‐based materials. In the TG 1% MAP 0.5% film, the feature observed around 2*θ* = 15° is more appropriately interpreted as part of the amorphous halo rather than as a distinct crystalline reflection. Similarly, pure TG exhibits broad peaks at 2*θ* = 21.4° and 28.9°, which have been associated with predominantly amorphous structures (Tabassum et al. 2024). Therefore, based on the present XRD data, it cannot be concluded that the incorporation of MAPs enhanced crystallinity in TG‐based films; rather, the diffraction pattern indicates that the matrix remained mainly amorphous after MAP incorporation. Comparable changes in diffraction behavior relative to unmodified TG films have also been reported for TG films containing clove essential oil (Tabassum et al. 2024).

The PAGE 1% MAP 0.5% film similarly exhibited a broadened, low‐intensity halo in the 2*θ* range of 17°–20°, further indicating a predominantly amorphous structure. Overall, both film systems displayed mainly amorphous organization, although slight differences in halo shape and position may reflect variations in molecular arrangement within the polymer matrices. These observations may be considered alongside the mechanical results, which showed higher elongation at break (EB) for TG‐based composites, while PAGE‐based films exhibited lower flexibility (*p* < 0.05). However, these mechanical differences cannot be directly attributed to increased crystallinity based on the present XRD data. Previous studies have also reported differences in structural organization between insoluble and soluble fractions of *Prunus domestica* gum (Sharma et al. 2020), although such comparisons should be interpreted cautiously.

### 3.9 Thermal Behavior

**FIGURE 5:**
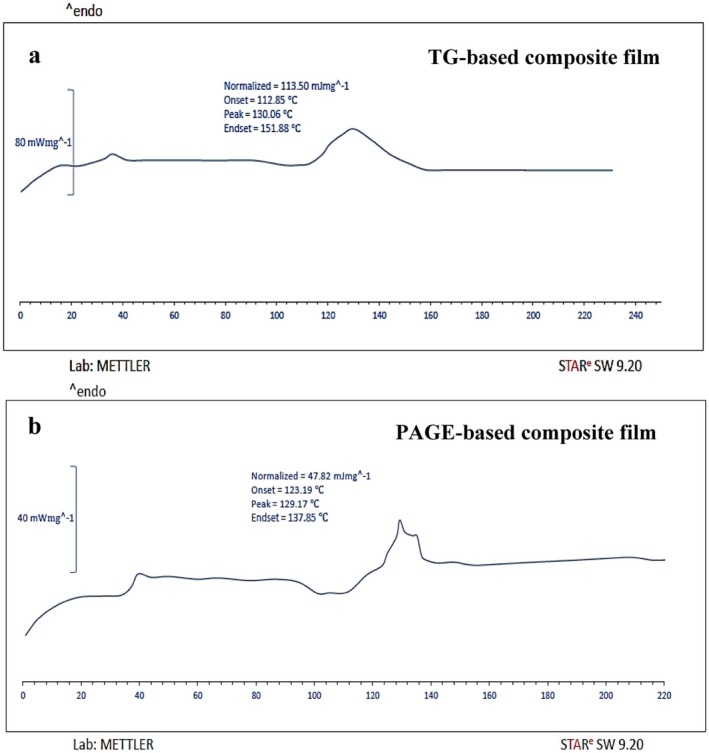
Differential scanning calorimetry of “TG 1% MAP 0.5%” (a) and “PAGE 1% MAP 0.5% (b)”.

The DSC thermograms of the TG 1% MAP 0.5% and PAGE 1% MAP 0.5% films are shown in Figure 5a,b. The TG‐based film exhibited an endothermic peak at 130.06°C with a normalized enthalpy of +113.50 mJ/mg, which is commonly associated with the evaporation of bound and residual water present in polysaccharide‐based matrices (Shahvalizadeh et al. 2021). Similarly, the PAGE‐based film showed a comparable endothermic transition at 129.17°C but with a lower enthalpy value (+47.82 mJ/mg). These endothermic events reflect differences in the quantity or state of water associated with each film rather than distinct melting transitions.

The lower enthalpy observed for the PAGE film indicates that less energy was required for moisture release during heating, whereas the higher enthalpy of the TG film suggests a greater amount of water retained within its matrix. These differences are consistent with the comparatively denser morphology observed in TG‐based films; however, detailed interpretation of polymer–water interactions should be made cautiously, particularly because only the instrument‐generated output plots were available for analysis.

### 3.10 Antibacterial Properties of Films

The antibacterial efficacy of the TG 1% MAP 0.5% and PAGE 1% MAP 0.5% films was assessed against four bacterial strains: *Escherichia coli* , *Staphylococcus aureus* , *Bacillus cereus* , and *Pseudomonas aeruginosa* using the agar diffusion method (Table 6). Both composite films exhibited inhibitory zones, with the TG‐based system showing consistently larger inhibition zones than the PAGE‐based counterpart. While the observed difference against *B. cereus* (20.00 ± 0.32 mm for TG vs. 19.00 ± 0.10 mm for PAGE) is statistically significant (*p* < 0.05), the numerical difference is small; thus, its practical significance regarding food safety efficacy should be interpreted with caution. The antibacterial performance is primarily attributed to the incorporated MAP fraction, which contains bioactive marine sulfated polysaccharides (fucoidan‐rich fractions) with established antimicrobial functions. The consistently larger inhibition zones observed for the TG‐based system suggest that the structural properties and film‐forming ability of the TG matrix may better facilitate the localized exposure or surface‐availability of these MAP bioactives compared to the PAGE matrix. While TG is a recognized hydrocolloid matrix with favorable structural properties (Bahrami, Nateghi, Rashidi, Nobandegani, and Ghorbanpour 2025), its role here likely relates to modulating the efficiency of the MAP fraction at the film‐agar interface rather than acting as a primary antimicrobial agent. In comparison, the PAGE‐based films demonstrated moderate activity, consistent with earlier findings by Salarbashi et al. (2021), which indicated that PAGE‐derived materials exhibit strain‐dependent sensitivity, showing stronger effects against Gram‐positive bacteria such as *S. aureus* than against Gram‐negative species. Furthermore, the inhibition zones reported here (up to 20 mm) are at the upper end of, and in some cases slightly higher than, those reported for other seaweed‐polysaccharide‐based films; for instance, composite films incorporating similar algal extracts or fucoidan fractions often produce inhibition zones in the range of 12–18 mm under comparable assay conditions (Hadi et al. 2022). It is important to note that high‐molecular‐weight polysaccharides such as gums and fucoidans generally exhibit limited diffusibility in agar media. Consequently, these results likely reflect localized surface interactions rather than diffusion‐driven release. In the absence of control films (neat TG and PAGE without MAP), it remains difficult to fully deconvolute the specific baseline contribution of the matrix from that of the bioactive additives. Therefore, these results should be viewed as a comparative assessment of how different matrix environments influence the performance of incorporated marine polysaccharides.

**TABLE 6:** Inhibition zone of composite films containing TG, PAGE, and MAPs (rich in fucoidan).

Sample	Inhibition zone (mm)			
	*E. coli*	*S. aureus*	*B. cereus*	*P. aeruginosa*
TG 1% MAP 0.5%	20.00 ± 0.08^a^	14.00 ± 0.15^a^	20.00 ± 0.32^a^	15.00 ± 0.34^a^
PAGE 1% MAP 0.5%	15.00 ± 0.24^b^	12.00 ± 0.20^b^	19.00 ± 0.10^b^	14.00 ± 0.29^b^

*Note:* Different small superscripts indicate significant difference between the columns (*p* < 0.05).

## 4 Conclusion

Biocomposite films were successfully fabricated from tragacanth gum (TG), *Prunus armeniaca* L. exudate gum (PAGE), and marine algal polysaccharides (MAPs). Among the investigated formulations, films containing 1% TG or 1% PAGE combined with 0.5% MAPs showed balanced physicochemical and functional characteristics relevant to potential food‐packaging applications.

Regarding solubility, the TG 1% MAP 0.5% film exhibited the lowest value (22.00% ± 0.23%), while TG 0.5% MAP 1% showed 23.00% ± 0.40%. In comparison, the corresponding PAGE 1% MAP 0.5% film presented a solubility of 25.00% ± 0.40%, indicating that the TG‐based formulation had lower water sensitivity under the tested conditions. For moisture barrier performance, the TG 1% MAP 0.5% film exhibited the lowest water vapor permeability (0.40 ± 0.00 g·m/(m^2^·s·Pa)), compared with PAGE 1% MAP 0.5% (0.70 ± 0.04 g·m/(m^2^·s·Pa)), indicating higher resistance to water vapor transmission. Mechanical testing showed that the TG 1% MAP 0.5% film had a tensile strength of 60.00 ± 0.34 MPa, whereas the PAGE 1% MAP 0.5% film showed 35.60 ± 2.00 MPa. Among the selected formulations, the PAGE 1% MAP 0.5% film exhibited higher elongation at break than the TG 1% MAP 0.5% film, indicating greater flexibility, whereas the TG‐based formulation provided greater mechanical resistance.

Structural analyses supported these results. SEM and XRD observations indicated a more homogeneous structure in TG‐based films, which may contribute to reduced diffusion pathways for water vapor. DSC analysis indicated differences in thermal behavior between the films, with the TG‐based film showing a higher endothermic enthalpy, which may reflect stronger water association and intermolecular interactions within the matrix.

Antibacterial testing demonstrated that TG 1% MAP 0.5% produced inhibition zones of 20.00 ± 0.08 mm ( *E. coli* ), 14.00 ± 0.15 mm ( *S. aureus* ), 20.00 ± 0.32 mm ( *B. cereus* ), and 15.00 ± 0.34 mm ( *P. aeruginosa* ). These values were larger than those obtained for PAGE 1% MAP 0.5% (e.g., 15.00 ± 0.24 mm for *E. coli* ), indicating stronger antibacterial activity under the conditions of the agar diffusion assay. Overall, among the tested formulations, the TG 1% MAP 0.5% film exhibited the lowest water vapor permeability and solubility together with the highest tensile strength and larger antibacterial inhibition zones. These characteristics indicate the potential of TG–MAP composite films as bio‐based materials for food‐packaging applications. Further studies, including biodegradability evaluation and long‐term storage performance, are required to assess their suitability for sustainable packaging systems.

## Author Contributions

**Sahba Bahrami Freadooni:** writing – original draft, formal analysis. **Leila Nateghi:** methodology, writing – review and editing, software. **Mansooreh Mazaheri:** validation, writing – review and editing. **Ladan Rashidi:** supervision, writing – review and editing, resources. **Mohammadreza Vafaee:** writing – review and editing.

## Funding

The authors have nothing to report.

## Conflicts of Interest

The authors declare no conflicts of interest.

## Supporting information


**Figure S1:** Representative linear regression plots of weight gain versus time for the WVP determination of (a) TG 1% MAP 0.5% and (b) PAGE 0.5% MAP 1% composite films. The plots demonstrate high linearity ($R^{2}>0.999$) over the 7‐h measurement period, confirming that the water vapor transmission reached a steady state in accordance with ASTM E96/E96M‐16.

## Data Availability

The data from the current study are available from the corresponding author upon reasonable request.

## References

[fsn372103-bib-0001] Bahrami, S. , L. Nateghi , and L. Rashidi . 2025. “Improvement of Oxidative Stability and Shelf Life of Beluga ( *Huso huso* ) Fillets Using Nanocomposite Films Constituted With *Prunus armeniaca* L. Gum Exudates (PAGE), Tragacanth Gum (TG), Fucoidan, and Zinc Oxide.” Food Chemistry: X 29: 102842. 10.1016/j.fochx.2025.102842. 40791880 PMC12337700

[fsn372103-bib-0002] Bahrami, S. , L. Nateghi , L. Rashidi , B. K. Nobandegani , and M. Ghorbanpour . 2025. “Evaluation of the Properties of Polysaccharides Extracted From Brown Macroalgae ( *Sargassum ilicifolium* ) by Methods of Conventional, Microwave, and Subcritical Water Extraction.” Innovative Food Science & Emerging Technologies 102: 103975. 10.1016/j.ifset.2025.103975.

[fsn372103-bib-0003] Brovko, O. , I. Palamarchuk , N. Gorshkova , and D. Chukhchin . 2024. “Investigation of Interpolymer Complexes of Fucoidan With Sodium Alginate in Solutions and Films.” Journal of Applied Phycology 37, no. 3: 539–551. 10.1007/s10811-024-03377-w.

[fsn372103-bib-0004] Carpintero, M. , I. Marcet , M. Rendueles , and M. Díaz . 2023. “Algae as an Additive to Improve the Functional and Mechanical Properties of Protein and Polysaccharide‐Based Films and Coatings: A Review of Recent Studies.” Food Packaging and Shelf Life 38: 101128. 10.1016/j.fpsl.2023.101128.

[fsn372103-bib-0005] Da Silva, M. G. A. , I. D. S. da Silva , A. K. Albuquerque , et al. 2025. “Valorization of Agro‐Industrial Residues: Structure–Property Relationships in Starch‐Based Films From Potato Peel Waste by Citric Acid Crosslinking and Kinetic Modeling of Gelatinization.” Materials Today Communications 48: 113486. 10.1016/j.mtcomm.2025.113486.

[fsn372103-bib-0006] Fan, Y. , J. Ren , X. Xiao , et al. 2025. “Recent Advances in Polysaccharide‐Based Edible Films/Coatings for Food Preservation: Fabrication, Characterization, and Applications in Packaging.” Carbohydrate Polymers 364: 123779. 10.1016/j.carbpol.2025.123779. 40484600

[fsn372103-bib-0007] Gohari, A. S. , L. Nateghi , L. Rashidi , and S. Berenji . 2024. “Preparation and Characterization of Sodium Caseinate‐Apricot Tree Gum/Gum Arabic Nanocomplex for Encapsulation of Conjugated Linoleic Acid (CLA).” International Journal of Biological Macromolecules 261: 129773. 10.1016/j.ijbiomac.2024.129773. 38296128

[fsn372103-bib-0008] Gomaa, M. , A. F. Hifney , M. A. Fawzy , and K. M. Abdel‐Gawad . 2018. “Use of Seaweed and Filamentous Fungus Derived Polysaccharides in the Development of Alginate‐Chitosan Edible Films Containing Fucoidan: Study of Moisture Sorption, Polyphenol Release, and Antioxidant Properties.” Food Hydrocolloids 82: 239–247. 10.1016/j.foodhyd.2018.03.056.

[fsn372103-bib-0009] Hadi, A. , A. Nawab , F. Alam , and K. Zehra . 2022. “Alginate/Aloe Vera Films Reinforced With Tragacanth Gum.” Food Chemistry: Molecular Sciences 4: 100105. 10.1016/j.fochms.2022.100105. 35769402 PMC9235049

[fsn372103-bib-0010] James, K. , H. Long , I. O'Hara , B. Williams , and L. Moghaddam . 2024. “Bio‐Based Film Development: Harnessing Alginate and Fucoidan Extracted From *Ascophyllum nodosum* With Glycerol and Choline Chloride‐Based Solvent.” Food Hydrocolloids 153: 110013. 10.1016/j.foodhyd.2024.110013.

[fsn372103-bib-0011] Khodaei, D. , K. Oltrogge , and Z. Hamidi‐Esfahani . 2020. “Preparation and Characterization of Blended Edible Films Manufactured Using Gelatin, Tragacanth Gum, and Persian Gum.” LWT 117: 108617. 10.1016/j.lwt.2019.108617.

[fsn372103-bib-0012] Liang, F. , C. Liu , J. Geng , et al. 2024. “Chitosan–Fucoidan Encapsulating Cinnamaldehyde Composite Coating Films: Preparation, pH‐Responsive Release, Antibacterial Activity and Preservation for Litchi.” Carbohydrate Polymers 333: 121968. 10.1016/j.carbpol.2024.121968. 38494223

[fsn372103-bib-0013] Mostafavi, F. S. , R. Kadkhodaee , B. Emadzadeh , and A. Koocheki . 2016. “Preparation and Characterization of Tragacanth–Locust Bean Gum Edible Blend Films.” Carbohydrate Polymers 139: 20–27. 10.1016/j.carbpol.2015.11.069. 26794942

[fsn372103-bib-0014] Nejatian, M. , S. Abbasi , and F. Azarikia . 2020. “Gum Tragacanth: Structure, Characteristics, and Applications in Foods.” International Journal of Biological Macromolecules 160: 846–860. 10.1016/j.ijbiomac.2020.05.214. 32474076

[fsn372103-bib-0015] Pirsa, S. , F. Mohtarami , and S. Kalantari . 2020. “Preparation of Biodegradable Composite Starch/Tragacanth Gum/Nanoclay Film and Study of Its Physicochemical and Mechanical Properties.” Chemical Review Letters 3: 98–103. 10.22034/CRL.2020.229483.1055.

[fsn372103-bib-0016] Pouralkhas, M. , M. Kordjazi , S. M. Ojagh , and O. A. Farsani . 2023. “Physicochemical and Functional Characterization of Gelatin Edible Film Incorporated With Fucoidan Isolated From *Sargassum tenerrimum* .” Food Science & Nutrition 11: 4124–4135. 10.1002/fsn3.3402. 37457150 PMC10345729

[fsn372103-bib-0017] Puhari, S. S. M. , S. Yuvaraj , V. Vasudevan , et al. 2022. “Isolation and Characterization of Fucoidan From Four Brown Algae and Study of the Cardioprotective Effect of Fucoidan From *Sargassum wightii* Against High Glucose‐Induced Oxidative Stress in H9c2 Cardiomyoblast Cells.” Journal of Food Biochemistry 46: e14412. 10.1111/jfbc.14412. 36121745

[fsn372103-bib-0018] Rashidi, L. 2022. “Standards and Guidelines for Testing Biodegradability of Bioplastic.” In Biodegradable Polymer‐Based Food Packaging, 297–325. Springer Nature. 10.1007/978-981-19-5743-7_15.

[fsn372103-bib-0019] Rezvankhah, A. , Z. Emam‐Djomeh , M. Safari , M. Salami , and G. Askari . 2022. “Investigating the Effects of Maltodextrin, Gum Arabic, and Whey Protein Concentrate on the Microencapsulation Efficiency and Oxidation Stability of Hemp Seed Oil.” Journal of Food Processing and Preservation 46: e16554. 10.1111/jfpp.16554.

[fsn372103-bib-0020] Roy, D. , M. K. A. Sobuj , M. S. Islam , et al. 2024. “Compositional, Structural, and Functional Characterization of Fucoidan Extracted From *Sargassum polycystum* Collected From Saint Martin's Island, Bangladesh.” Algal Research 80: 103542. 10.1016/j.algal.2024.103542.

[fsn372103-bib-0021] Salarbashi, D. , K. Jahanbin , M. Tafaghodi , and E. Fahmideh‐Rad . 2021. “ *Prunus armeniaca* Gum Exudates: An Overview on Purification, Structure, Physicochemical Properties, and Applications.” Food Science & Nutrition 9: 1240–1255. 10.1002/fsn3.2107. 33598208 PMC7866599

[fsn372103-bib-0022] Samani, M. A. , S. Babaei , M. Naseri , M. Majdinasab , and A. M. Nafchi . 2023. “Development and Characterization of a Novel Biodegradable and Antioxidant Film Based on Marine Seaweed Sulfated Polysaccharide.” Food Science & Nutrition 11: 3767–3779. 10.1002/fsn3.3361. 37457178 PMC10345713

[fsn372103-bib-0023] Shahvalizadeh, R. , R. Ahmadi , I. Davandeh , et al. 2021. “Antimicrobial Bio‐Nanocomposite Films Based on Gelatin, Tragacanth, and Zinc Oxide Nanoparticles – Microstructural, Mechanical, Thermo‐Physical, and Barrier Properties.” Food Chemistry 354: 129492. 10.1016/j.foodchem.2021.129492. 33756322

[fsn372103-bib-0024] Sharma, A. , P. R. Bhushette , and U. S. Annapure . 2020. “Purification and Physicochemical Characterization of *Prunus domestica* Exudate Gum Polysaccharide.” Carbohydrate Polymer Technologies and Applications 1: 100003. 10.1016/j.carpta.2020.100003.

[fsn372103-bib-0025] Sheybani, F. , L. Rashidi , L. Nateghi , M. Yousefpour , and S. K. Mahdavi . 2023a. “Application of Nanostructured Lipid Carriers Containing α‐Tocopherol for Oxidative Stability Enhancement of Camelina Oil.” Industrial Crops and Products 202: 117007. 10.1016/j.indcrop.2023.117007.

[fsn372103-bib-0026] Sheybani, F. , L. Rashidi , L. Nateghi , M. Yousefpour , and S. K. Mahdavi . 2023b. “Development of Ascorbyl Palmitate‐Loaded Nanostructured Lipid Carriers (NLCs) to Increase the Stability of Camelina Oil.” Food Bioscience 53: 102735. 10.1016/j.fbio.2023.102735.

[fsn372103-bib-0027] Tabassum, Z. , A. Babu , H. S. Ahmed , et al. 2024. “Development of Antibacterial Edible Films for Food Packaging Using Tragacanth Gum, Carrageenan, and Clove Essential Oil.” Journal of Applied Polymer Science 141: e55495. 10.1002/app.55495.

[fsn372103-bib-0028] Tabassum, Z. , M. Girdhar , A. Kumar , T. Malik , and A. Mohan . 2023. “ZnO Nanoparticles‐Reinforced Chitosan‐Xanthan Gum Blend Novel Film With Enhanced Properties and Degradability for Application in Food Packaging.” ACS Omega 8: 31318–31332. 10.1021/acsomega.3c03763. 37663466 PMC10468839

[fsn372103-bib-0029] Taktak, F. F. , and H. N. Kaya . 2025a. “Biodegradable PVA/Chitosan‐Based Films Enriched With Rose Hip Extract and Seed Oil: Investigation of the Influence of Tragacanth Gum Ratio on Functional Properties and Its Application in Cherry Preservation.” International Journal of Biological Macromolecules 307, no. Part 1: 141023. 10.1016/j.ijbiomac.2025.141023. 40010468

[fsn372103-bib-0030] Taktak, F. F. , and H. N. Kaya . 2025b. “Enhanced Antibacterial Activity and Biodegradability of PVA/Chitosan/Gum Tragacanth Films Enriched With ZnO and Safflower Oil.” Journal of Macromolecular Science, Part B: 1–24. 10.1080/00222348.2025.2584766.

[fsn372103-bib-0031] Tonyali, B. , S. Cikrikci , and M. H. Oztop . 2018. “Physicochemical and Microstructural Characterization of Gum Tragacanth Added Whey Protein‐Based Films.” Food Research International 105: 1–9. 10.1016/j.foodres.2017.10.071. 29433188

[fsn372103-bib-0032] Ummat, V. , S. P. Sivagnanam , S. Rameshkumar , et al. 2024. “Sequential Extraction of Fucoidan, Laminarin, Mannitol, Alginate and Protein From Brown Macroalgae Ascophyllum Nodosum and *Fucus vesiculosus* .” International Journal of Biological Macromolecules 256: 128195. 10.1016/j.ijbiomac.2023.128195. 38008143

[fsn372103-bib-0033] Zhang, K. , Q. Chen , J. Xiao , et al. 2023. “Physicochemical and Functional Properties of Chitosan‐Based Edible Film Incorporated With *Sargassum pallidum* Polysaccharide Nanoparticles.” Food Hydrocolloids 138: 108476. 10.1016/j.foodhyd.2023.108476.

