# Supplementary material for: Edible Biocomposite Films From Tragacanth and Apricot Gums Reinforced With Marine Algal Polysaccharides: Development, Characterization, and Antibacterial Efficacy
Source: Food Sci Nutr. 2026 Jul 10;14(7):e72103. doi: 10.1002/fsn3.72103 (PMC13351608; doi:10.1002/fsn3.72103)
Supplement: Supplementary file 1 — Figure S1: Representative linear regression plots of weight gain versus time for the WVP determination of (a) TG 1% MAP 0.5% and (b) PAGE 0.5% MAP 1% composite films. The plots demonstrate high linearity (R2>0.999) over the 7‐h measurement period, confirming that the water vapor transmission reached a steady state in accordance with ASTM E96/E96M‐16. [file FSN3-14-e72103-s001.docx]

**
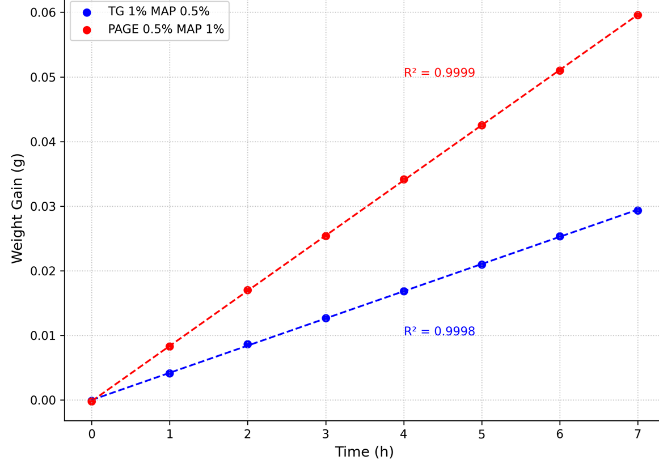
**

**Figure S1.** Representative linear regression plots of weight gain versus time for the WVP determination of (a) TG 1% MAP 0.5% and (b) PAGE 0.5% MAP 1% composite films. The plots demonstrate high linearity ($R^{2}>0.999$) over the 7-hour measurement period, confirming that the water vapor transmission reached a steady state in accordance with ASTM E96/E96M-16.
